# Combination of ERK2 inhibitor VX-11e and voreloxin synergistically enhances anti-proliferative and pro-apoptotic effects in leukemia cells

**DOI:** 10.1007/s10495-019-01564-6

**Published:** 2019-09-03

**Authors:** Ewa Jasek-Gajda, Halina Jurkowska, Małgorzata Jasińska, Jan A. Litwin, Grzegorz J. Lis

**Affiliations:** 1 Department of Histology, Jagiellonian University Medical College, Kopernika 7, 31034 Kraków, Poland; 2 Department of Medical Biochemistry, Jagiellonian University Medical College, Kraków, Poland

**Keywords:** VX-11e, Voreloxin, Leukemia cell lines, Apoptosis, Cell cycle

## Abstract

ERK1/2 inhibitors are new promising anticancer drugs. The aim of this study was to investigate the effect of the combination of ERK2 inhibitor VX-11e and voreloxin on MOLM-14, K562, REH and MOLT-4 leukemia cell lines. We found that VX-11e alone and in combination with voreloxin significantly decreased ERK activation in all cell lines tested. To evaluate the interactions of the drugs, cells were treated for 24 h with VX-11e or voreloxin alone and in combination at fixed ratios based on IC_50_ values. The combinatorial effects of both drugs were synergistic over a wide range of concentrations in MOLM-14, REH and MOLT-4 cell lines. In K562 cells, three effects were found to be additive, one antagonistic and only one synergistic. The results showed that incubation with both VX-11e and voreloxin inhibited the growth of leukemia cells, affected cell cycle and induced apoptosis. Furthermore, the molecular mechanism of these effects might be attributed to an increased expression of p21 and a decreased expression of survivin and NF-κB in all cell lines tested except from K562 cells. In conclusion, combination of VX-11e and voreloxin can exert a synergistic anticancer effect in leukemia cells.

## Introduction

Extracellular signal-regulated kinases (ERKs) participate in the downstream signaling of Ras/Raf/mitogen-activated protein kinase (MEK)/ERK (Ras/Raf/MEK/ERK) pathway as key regulators that control proliferation, differentiation and cell survival. ERK1/2 phosphorylates a number of downstream transcription factors, including nuclear factor-κB (NF-κB) and alters the expression of many proteins regulating cell cycle and apoptosis, such as p21 and survivin. [1–4]. Dysregulated signaling of the Ras/Raf/MEK/ERK cascade has been observed in many malignancies, including leukemias [2, 5–7], and these findings have stimulated an interest in developing specific inhibitors of this pathway [8–11]. However, since the proteins of the Ras/Raf/MEK/ERK pathway are involved in many cellular processes, it might be more effective to directly inhibit ERK protein bypassing upstream signaling components. Moreover, ERK inhibitors could be less sensitive to drug resistance mechanism than other inhibitors of the upstream molecules in Ras/Raf/MEK/ERK pathway [12]. Thus, targeting ERK isoforms directly is considered to be more advantageous in cancer treatment.

VX-11e is a potent and selective ERK2 inhibitor that has been shown to reduce tumour growth in melanoma xenograft models and to decrease proliferation and viability in various human cancer cell lines [13–15]. Furthermore, VX-11e has been found to exert synergistic effect when used in combination with megestrol in T cell prolymphocytic leukemia cells [16].

Voreloxin is a first-in-class topoisomerase II inhibitor which causes cell cycle arrest and apoptosis in leukemia cells [17–19]. It has been studied as single agent and in combination with other targeted drugs in the treatment of acute myeloid leukemia (AML) [18, 20, 21]. In our previous study, we have demonstrated that voreloxin acts in synergy with MEK inhibitor TAK-733 in HL60 cells [11].

The aim of the present study was to investigate whether the combination of VX-11e, an ERK2 inhibitor, with voreloxin exerts synergistic effects on human leukemia cells, and to examine possible mechanisms for such synergy.

## Materials and methods

### Drugs

VX-11e and voreloxin were purchased form Selleck Chemicals (Selleckchem, Houston, TX, USA). Stock solutions of drugs were dissolved in DMSO, aliquoted and kept frozen at − 80 °C until use.

### Cell culture

MOLT-4 (human acute T-cell lymphoblastic leukemia, ALL), REH (human acute B-cell lymphoblastic leukemia, ALL) and MOLM-14 (human acute myeloblastic leukemia, AML) cell lines were obtained from the German Collection of Microorganisms and Cell Cultures (DSMZ, Braunschweig, Germany). K562 (human chronic myeloid leukemia, CML) cells were purchased from the European Collection of Cell Cultures (ECACC, Salisbury, UK). All cell lines were maintained in RPMI-1640 GlutaMax medium supplemented with 10% fetal bovine serum (FBS) containing 100 U/ml penicillin, and 100 µg/ml streptomycin (all reagents from Life Technologies, Carlsbad, CA, USA). Cells were cultured at 37 °C in a humidified 5% CO_2_ atmosphere.

### Cell proliferation assay

The Muse Ki67 Proliferation Kit was used to assess cell proliferation according to the manufacturer’s instructions (Merck Millipore, Billerica, MA, USA). Briefly, 5 × 10^5^ cells were fixed, permeabilized and stained with an antibody against Ki-67-PE for 30 min at room temperature in the dark and quantified using Muse Cell Analyzer. The proliferating (Ki67-positive) cells were quantified using Muse analysis software. The concentrations of drugs required to inhibit 50% of cell growth (IC_50_) were calculated using Prism 5.0 software (GraphPad Software, Inc. La Jolla, CA, USA). The relative cell growth was calculated as the percentage of untreated cells.

### Drug interaction experiments

To evaluate the interactions of the drugs, cell lines were treated for 24 h with VX-11e or voreloxin alone and in combination at fixed ratios based on IC_50_ values. The potential synergistic effect of drugs was analyzed by calculating the combination index (CI) and fraction affected (Fa) based on the Chou–Talalay method [22] using CompuSyn software (CompuSyn Inc. Paramus, NJ, USA). CI values ˂ 0.9 were considered as synergistic, values ˃ 1.1 as antagonistic, and values 0.9–1.1 as additive.

### ERK activity assay

The Muse MAPK Activation Dual Detection Kit (Merck Millipore), including phospho-specific anti-phospho-ERK1/2 (Thr202/Tyr204,Thr185/Tyr187)-Phycoerythrin and anti-ERK1/2-PECy5-conjugated antibodies, was used to measure ERK expression in cells. Briefly, 10 × 10^5^ cells were washed with PBS and fixed for 5 min on ice. Then, the cells were treated with permeabilization buffer for 5 min on ice and incubated with solution of antibodies for 30 min at room temperature in the dark. Cells were quantified using Muse Cell Analyzer. ERK activation was calculated as the percentage of ERK phosphorylation relative to the total ERK expression in the cell population.

### Cell-cycle analysis

Cell cycle distribution was determined using the Muse Cell Cycle Kit (Merck Millipore) according to the manufacturer’s instructions. The assay is based on the measurement of DNA content in nuclei labeled with propidium iodide (PI). Briefly, 5 × 10^5^ cells were harvested and fixed with 70% ice cold ethanol at − 20 °C for 18 h. After washing with PBS, cell pellets were resuspended in 200 μl of Cell Cycle Reagent and incubated for 30 min at room temperature in the dark. Cells were analyzed by Muse Cell Analyzer and the cell cycle phase distribution was quantified using Muse analysis software.

### Apoptosis assay

Apoptotic cells were analyzed using Muse Annexin V and Dead Cell Kit (Merck Millipore) according to the previously described protocol (11). This assay utilizes Annexin V to detect phosphatidylserine on the external membrane leaflet of apoptotic cells and dead cell marker, 7-AAD, as an indicator of cell membrane integrity. Briefly, 1 × 10^5^ cells were resuspended in culture medium containing 1% FBS and incubated with Muse Annexin V and Dead Cell Reagent for 20 min at room temperature in the dark. Cells were quantified using Muse Cell Analyzer and Muse analysis software.

### Western blotting

Cells were lysed in radioimmunoprecipitation assay (RIPA) buffer (Sigma-Aldrich, Poznan, Poland) containing 1% Protease Inhibitor Coctail (Roche Diagnostic, Basel, Switzerland) followed by centrifugation at 20,000×*g* for 15 min at 4 °C. Protein concentration was determined by the bicinchoninic acid (BCA) method using BCA Protein Assay Kit (Thermo Scientific/Pierce Biotechnology, Rockford, IL, USA) with bovine serum albumin (Merck Millipore) as a standard. Equal amounts of proteins (40 µg) were separated by sodium dodecyl sulfate–polyacrylamide gel electrophoresis (SDS-PAGE) on 4–20% Mini-Protean TGX precast gels (Bio-Rad Laboratories, Hercules, CA, USA) and transferred to polyvinylidene-fluoride membranes (PVDF) (Bio-Rad) at 100 V for 2 h. After incubation with blocking reagent (Bio-Rad), the membranes were probed with the following primary antibodies: anti-survivin (1:1000, Cell Signaling Technology (CST), Danvers, MA, USA), anti-p21 (1:1000, CST), anti-NF-κB p105/p50 (1:400, Abcam, Cambridge, UK) and anti-β-actin (1:1000, CST) overnight at 4 °C. After washing, the membranes were incubated at room temperature for 1 h with secondary goat anti-rabbit antibody conjugated with horseradish peroxidase (1:10,000, Bio-Rad). The protein bands were visualized with the Amplified Opti-4CN substrate kit (Bio-Rad) according to the manufacturer’s instructions. The relative optical density of blotting bands was quantified using ChemiDoc MP Imaging System (Bio-Rad). β-actin was used as the internal control.

### Confocal microscopy

Cytospin smears of control and treated cells were fixed with 4% buffered paraformaldehyde for 5 min at room temperature. After washing with PBS, cells were pre-incubated in primary antibody dilutor (PAD) comprising 10% normal goat serum, 0.1% bovine serum albumin, 0.1% Triton X-100, 0.05% thimerosal and 0.01% sodium azide (all reagents from Sigma) for 30 min at room temperature. Primary rabbit anti- NF-κB p105/p50 monoclonal antibody (Abcam; diluted 1:200 in PAD) was applied for an overnight incubation at room temperature. Following a wash with PBS, cells were incubated with secondary Cy3-conjugated goat anti-rabbit IgG antibody (Jackson ImmunoResearch, West Grove, PA, USA; diluted 1:500 in PAD) for 1 h in the dark. Cells were then rinsed with PBS and stained with Hoechst 33342 (Sigma; 2.5 μg/ml in PBS) for 5 min. Images were obtained by confocal microscopy (Olympus FluoView 1200 on inverted stand IX83; Olympus, Tokyo, Japan). Sixty-times magnification immersion objective (NA = 1.4) was used and helium-neonium laser (453 nm) and diode laser (405 nm) were applied to excite red (Cy3) and blue (Hoechst) fluorescence, respectively. The stacks of optical sections were acquired and further processed with Olympus FV10 software. For quantification, fields were chosen arbitrarily and the number of NF-κB positive dots per nucleus was determined in 50 cells per line/treatment using NIH ImageJ software (http://rsb.info.nih.gov/ij/).

### Statistical analysis

The results are expressed as mean ± standard deviation (SD) of three independent experiments. Statistical analysis for differences among groups was performed by Mann–Whitney test, followed by Tukey’s tests for multiple comparisons, with *p *< 0.05 considered as statistically significant. Data were analyzed using the Prism 5.0 software.

## Results

### VX-11e and voreloxin inhibited leukemia cell proliferation

**Fig. 1:**
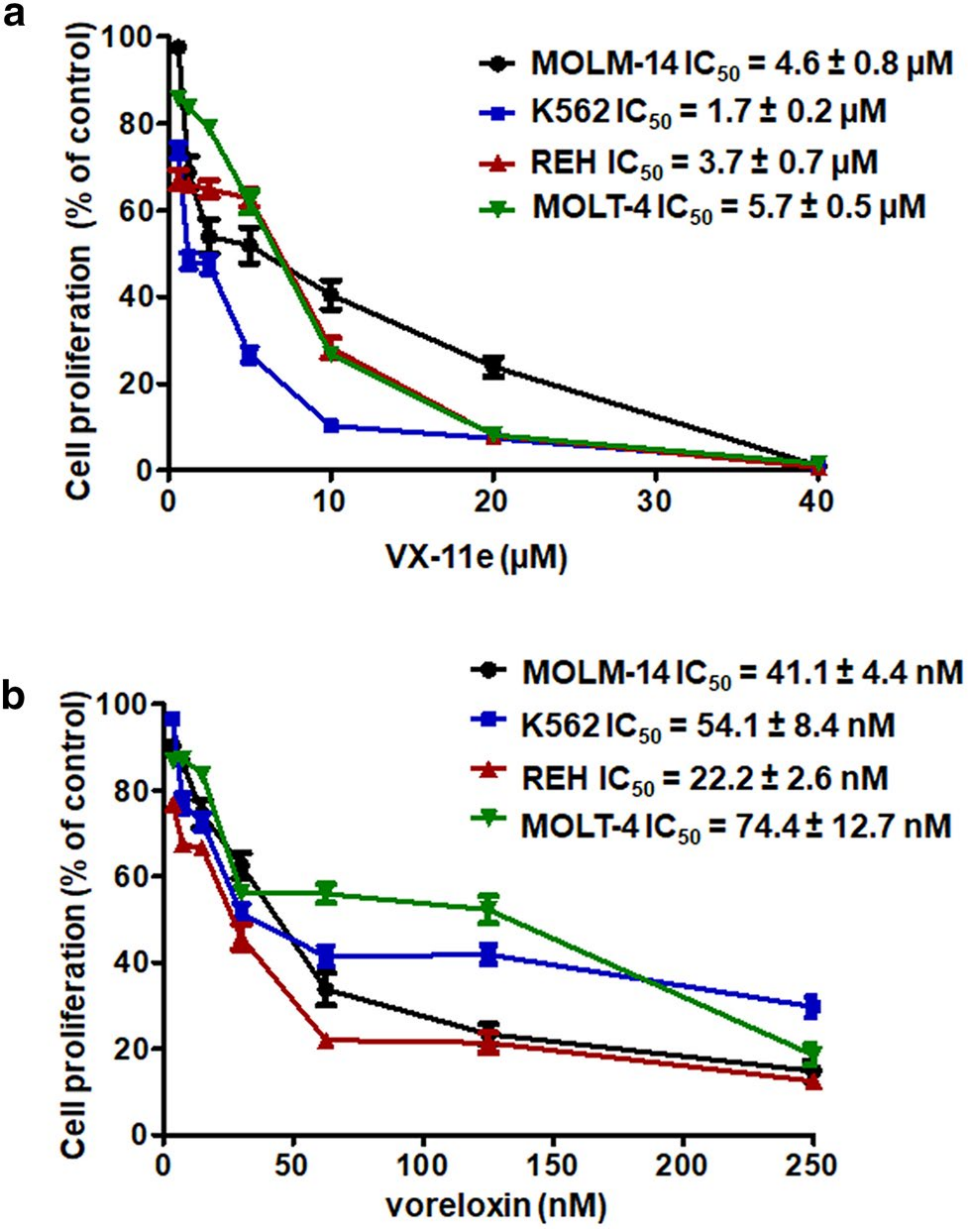
VX-11e (**a**) and voreloxin (**b**) inhibited leukemia cell proliferation. MOLM-14, K562, REH and MOLT-4 cells were incubated for 24 h with increasing concentrations of VX-11e (VX) or voreloxin (VOR). The percentages of proliferating cells and the IC_50_ values of each drug were determined by the Muse Ki67 Proliferation Kit. Each value is the mean ± SD of three independent experiments

MOLM-14, K562, REH and MOLT-4 cell lines were exposed to increasing concentrations of VX-11e (0.625 to 40 µM) and voreloxin (3.75 to 250 nM) for 24 h. The cell proliferation was inhibited in a dose-dependent manner. The IC_50_ values ranged from 1.7 ± 0.2 µM in K562 cells to 5.7 ± 0.5 µM in MOLT-4 cells for VX-11e and from 22.2 ± 2.6 nM in REH cells to 74.4 ± 12.7 nM in MOLT-4 cells for voreloxin (Fig. 1). These results show that K562 cells were the most sensitive to VX-11e and REH cells to voreloxin while MOLT-4 cells were the least sensitive to both drugs.

### Synergistic anti-proliferative effects of VX-11e and voreloxin

**Fig. 2:**
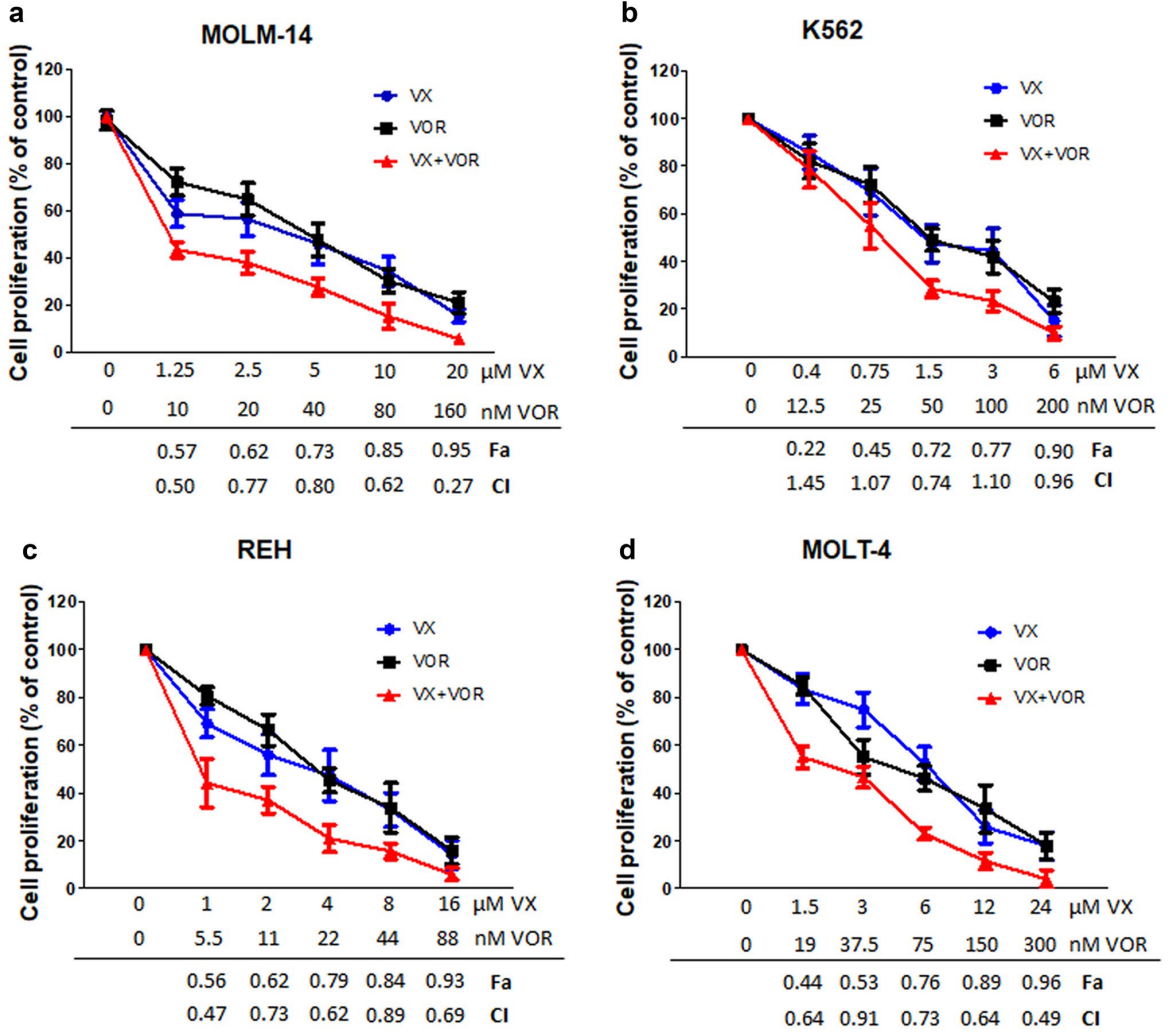
Synergistic anti-proliferative effects of VX-11e and voreloxin. **a** MOLM-14, **b** K562, **c** REH and **d** MOLT-4 cells were incubated for 24 h with the constant ratio dose at the IC_50_ values of VX-11e (VX) and voreloxin (VOR). The CI and Fa values were calculated using the Chou-Talalay method described in ″Materials and methods″. Each value is the mean ± SD of three independent experiments

For combination studies, VX-11e and voreloxin were used at the fixed ratio of their IC_50_ values. The combination index (CI) and fraction affected (Fa) were calculated to analyze the drug interaction (synergistic, additive or antagonistic). The combinations were synergistic over the wide range of concentrations in MOLM-14, REH and MOLT-4 cell lines, with the lowest CI of 0.27 and Fa of 0.95 in MOLM-14 cells (Fig. 2a, c and d). In K562 cells, three combinations were found to be additive (CI ranged from 0.96 to 1.45), one slight antagonistic (CI 1.07) and one synergistic (CI 0.74, Fa = 0.72) (Fig. 2b). Our data suggest that the combinatorial effects of VX-11e and voreloxin could be synergistic, additive or antagonistic, depending on drug concentration and cell line used. Since our aim was to achieve maximal effect of the drugs tested on leukemia cells, the combinations which generated the lowest CI values with Fa > 0.7 were used in further experiments: 20 µM of VX-11e and 160 nM of voreloxin in MOLM-14 cells, 1.5 µM of VX-11e and 50 nM of voreloxin in K562 cells, 4 µM of VX-11e and 22 nM of voreloxin in REH cells, and 24 µM of VX-11e and 300 nM of voreloxin in MOLT-4 cells.

### VX-11e alone and in combination with voreloxin decreased ERK activation

Leukemia cell lines were treated with VX-11e and voreloxin alone or in combination for 24 h.

**Fig. 3:**
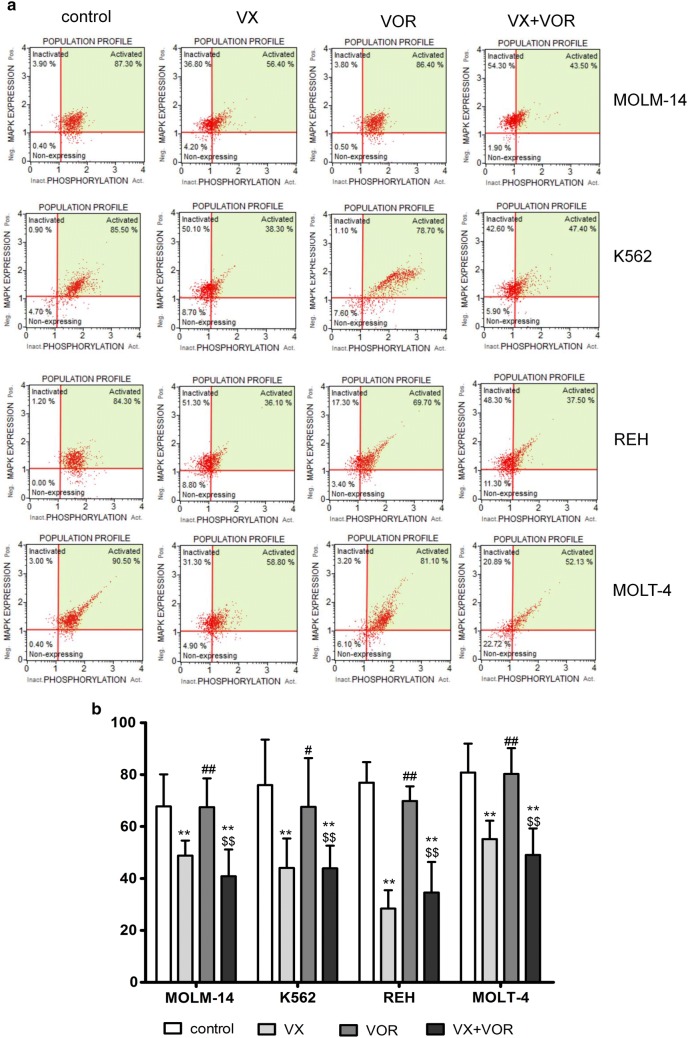
VX-11e alone and in combination with voreloxin decreased ERK activation. MOLM-14, K562, REH and MOLT-4 cells were incubated for 24 h with VX-11e and voreloxin alone or in combination. ERK activity was determined using the Muse MAPK Activation Dual Detection Kit. **a** Representative dot plots and **b** graph of ERK activation in MOLM-14, K562, REH and MOLT-4 cell lines. Each value is the mean ± SD of three independent experiments. *(p < 0.05), **(p < 0.01) versus control; #(p < 0.05), ^##^(p < 0.01) versus VX-11e; ^$^(p < 0.05), ^$$^(p < 0.01) versus voreloxin

After treatment with VX-11e alone and in combination with voreloxin, percentages of ERK activated cells were significantly reduced and reached the lowest relative level in REH cells (28.4% ± 7.1%). Voreloxin alone had no significant effect on ERK activation levels compared to control cells (Fig. 3a and b). These results indicate that VX-11e is highly effective for inhibition of ERK in leukemia cells.

### Combination of VX-11e and voreloxin induced cell-cycle arrest and apoptosis

**Fig. 4:**
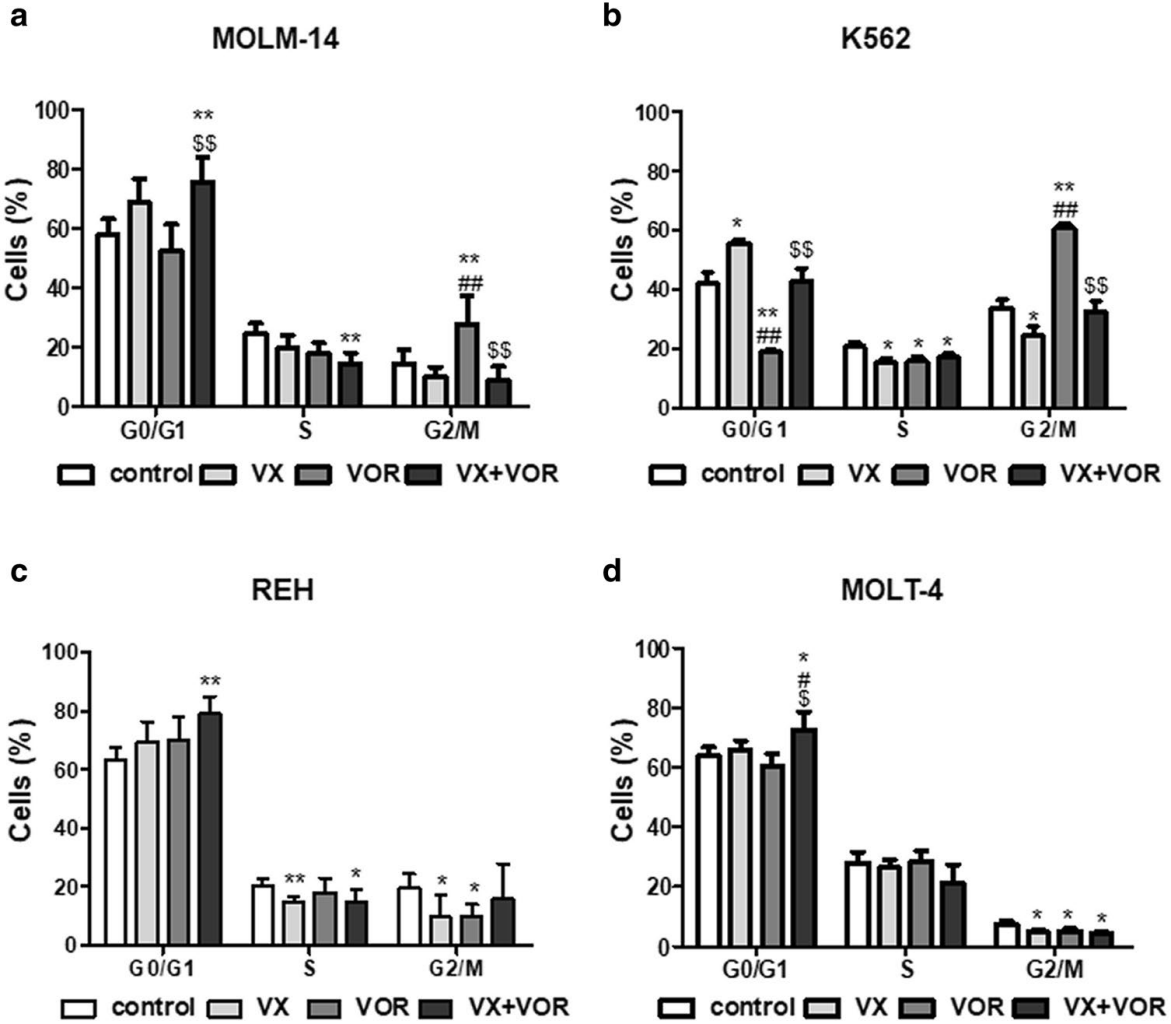
Combination of VX-11e and voreloxin induced cell-cycle arrest. **a** MOLM-14, **b** K562, **c** REH and **d** MOLT-4 cells were incubated for 24 h with VX-11e (VX) and voreloxin (VOR) alone or in combination. Cell cycle distribution was determined using the Muse Cell Cycle Kit. Each value is the mean ± SD of three independent experiments. *(p < 0.05), **(p < 0.01) versus control; ^#^(p < 0.05), ^##^(p < 0.01) versus VX-11e; ^$^(p < 0.05), ^$$^(p < 0.01) versus voreloxin

To further determine the potential synergistic effect of drug combination, we also analyzed the effect of drugs on the cell cycle and apoptosis. After treatment with VX-11e alone, the percentage of cells in G0/G1 phase was significantly increased in K562 cells, with a corresponding decrease of cells in the S and G2/M phases (Fig. 4b). Voreloxin alone did not significantly influence the cell cycle in studied cell lines, however, in K562 cells it caused an accumulation of cells in G2/M phase and a concomitant decrease in G0/G1 phase. The combination of VX-11e and voreloxin significantly induced cell cycle arrest in G0/G1 phase in all cell lines compared with the untreated control except K562 cells (Fig. 4d). As compared to the effect of voreloxin alone, the combined treatment caused further increase in the percentage of cells in G0/G1 phase in all cell lines except REH cells (Fig. 4c), while the percentage of cells in G2/M phase was significantly decreased in MOLM-14 and K562 cells (Fig. 4a and b).

**Fig. 5:**
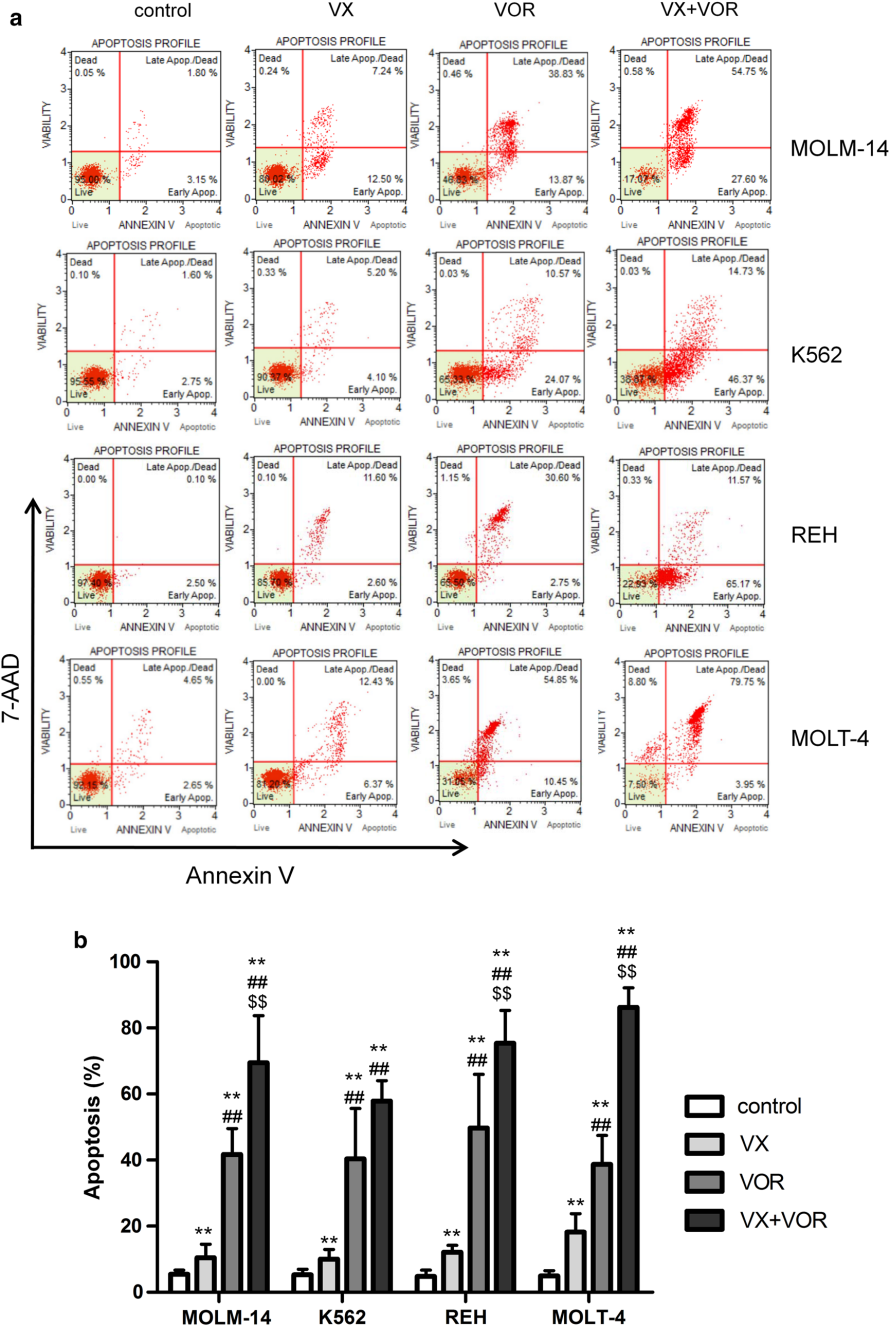
Combination of VX-11e and voreloxin enhanced apoptosis in leukemia cells. MOLM-14, K562, REH and MOLT-4 cells were incubated for 24 h with VX-11e (VX) and voreloxin (VOR) alone or in combinations. **a** Representative dot plots of Annexin V/7-AAD apoptotic assay and **b** graph showing the percentage of apoptotic cells. Each value is the mean ± SD of three independent experiments. *(p < 0.05), **(p < 0.01) versus control; ^#^(p < 0.05), ^##^(p < 0.01) versus VX-11e; ^$^(p < 0.05), ^$$^(p < 0.01) versus voreloxin

Treatment with VX-11e and voreloxin alone induced apoptosis in all cell lines (Fig. 5). The total apoptotic rate (early and late apoptosis) increased from 10% ± 3.3% in K562 cells to 18.2% ± 5.5% in MOLT-4 cells for VX-11e and from 38.7% ± 8.8% in MOLT-4 cells to 49.7% ± 16.2% for voreloxin. The combined treatment with VX-11e and voreloxin markedly potentiated apoptosis and the percentage of apoptotic cells ranged from 69.5% ± 14.2% in MOLM-14 cells to 86.2% ± 5.9% in MOLT-4 cells. This effect, however, was not observed in K562 cells (Fig. 5a and b). Hence, these results demonstrate that the induction of apoptosis may be related to cell cycle perturbations after combined treatment with VX-11e and voreloxin.

### VX-11e in combination with voreloxin increased p21 and reduced survivin and NF-κB protein levels

**Fig. 6:**
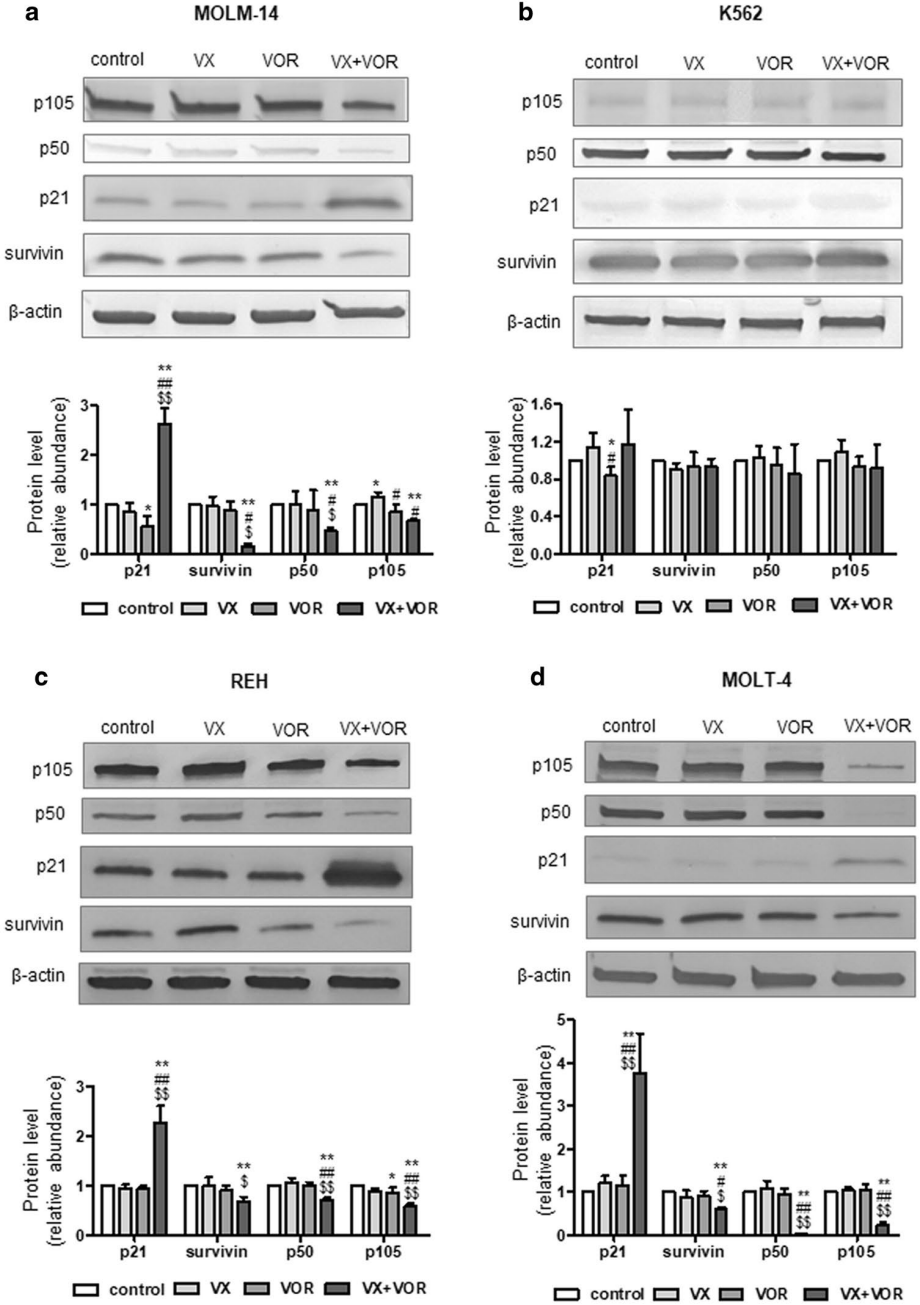
VX-11e in combination with voreloxin increased p21 and reduced survivin and NF-κB p105/p50 protein levels. **a** MOLM-14, **b** K562, **c** REH and **d** MOLT-4 cells were incubated for 24 h with VX-11e (VX) and voreloxin (VOR) alone or in combination. The expression of p21, survivin, p50 and p105 proteins was detected by Western blot. β-actin was used as a loading control. Quantification of the proteins was performed by densitometric analysis of the blots and normalized to the internal loading control. Each value is the mean ± SD of three independent experiments. *(p < 0.05), **(p < 0.01) versus control; ^#^(p < 0.05), ^##^(p < 0.01) versus VX-11e; ^$^(p < 0.05), ^$$^(p < 0.01) versus voreloxin

To elucidate the mechanism responsible for the anti-proliferative effects of VX-11e and voreloxin combination, we investigated proteins involved in the regulation of cell cycle and apoptosis by Western blotting. The amount of p21, an important checkpoint protein in G1 phase of the cell cycle, was markedly increased, whereas the level of anti-apoptotic survivin was significantly decreased in cells upon combined treatment with VX-11e and voreloxin, compared to untreated control and cells treated with either compound alone. Western blot analysis also showed a significant decrease in the level of NF-κB p105/p50 protein, a key regulator of pro-survival factors, compared to untreated control and cells treated with VX-11e or voreloxin alone. These effects were observed in all cell lines except from K562 cells (Fig. 6a–d). They might provide an explanation for the involvement of p21, survivin and NF-κB proteins in the synergistic anti-proliferative effect of VX11e and voreloxin combination.

### VX-11e in combination with voreloxin inhibited NF-κB translocation into the nucleus

**Fig. 7:**
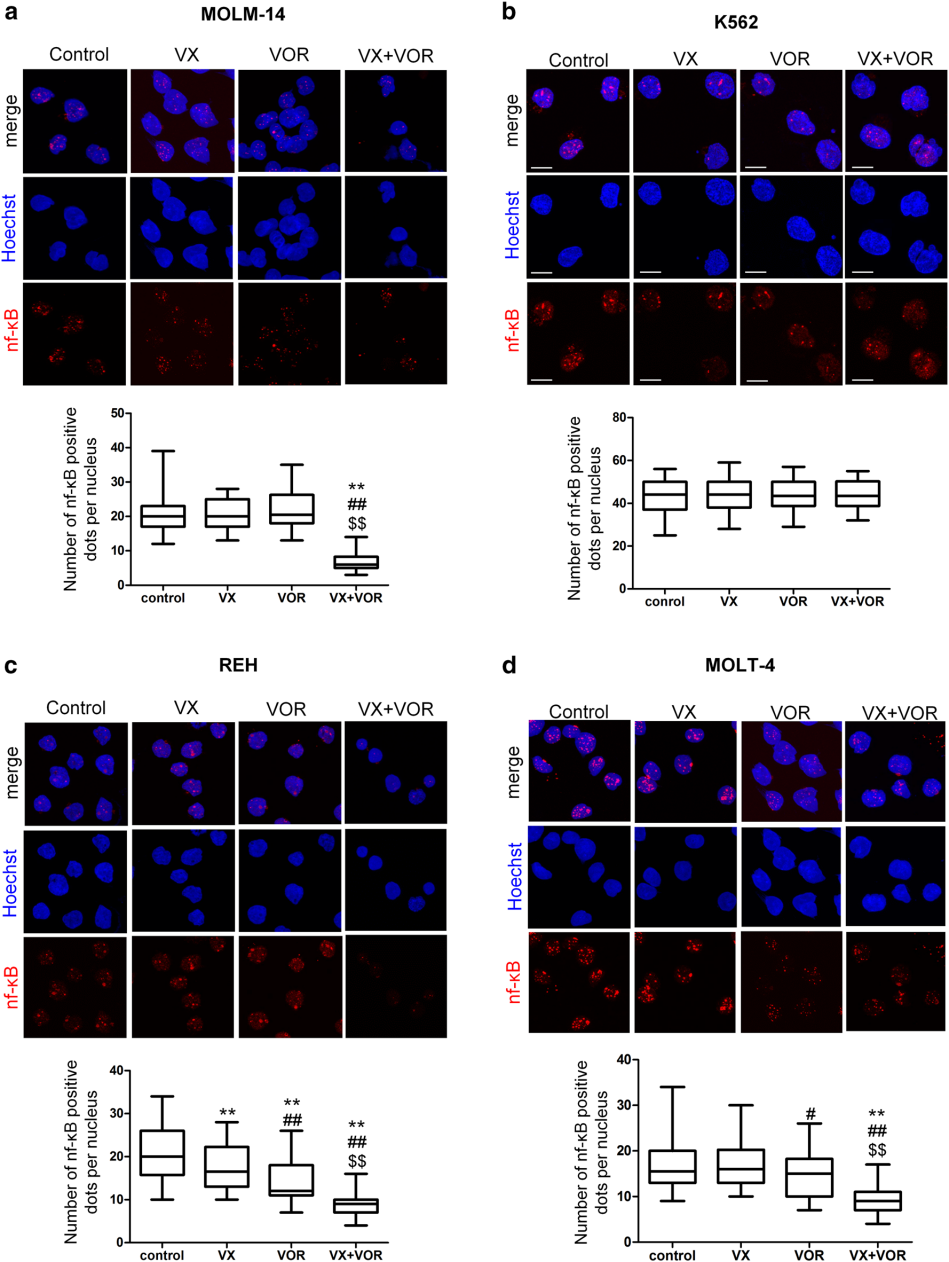
VX-11e in combination with voreloxin inhibited NF-κB translocation into the nucleus. **a** MOLM-14, **b** K562, **c** REH and **d** MOLT-4 cells were incubated for 24 h with VX-11e (VX) and voreloxin (VOR) alone or in combination. Cells were immunostained for NF-κB (red fluorescence) and the nuclei were stained blue with Hoechst 33342. Representative confocal images and box-and-whisker plots representing the number of NF-κB positive dots per nucleus from three independent experiments. Bar = 10 μm. The box, line and whiskers represent the quartiles, median and range of data (minimal and maximal values), respectively. *(p < 0.05), **(p < 0.01) versus control; ^#^(p < 0.05), ^##^(p < 0.01) versus VX-11e; ^$^(p < 0.05), ^$$^(p < 0.01) versus voreloxin (Color figure online)

In untreated control cells and in those treated with VX-11e and voreloxin alone, NF-κB was found to be distributed mainly in the nuclei, showing punctuated immunofluorescent pattern. Combinatorial treatment of cells with VX-11e and voreloxin showed significant decrease in the number of red fluorescent dots localized in the nuclei compared to untreated control and cells treated with either drug alone (Fig. 7a, c and d). These observations suggest that combinatorial treatment of cells with VX-11e and voreloxin inhibited nuclear translocation of NF-κB. However, this effect was not observed in K562 cells (Fig. 7b).

## Discussion

Combination of drugs directly targeting ERK kinases with traditional chemotherapeutics may provide new potential tools for cancer treatment. We recently reported that preincubation of HL60 myeloid leukemia cells with MEK inhibitor TAK-733 synergistically potentiated voreloxin-induced apoptosis [11]. In the present study, we have shown that ERK2 inhibitor VX-11e demonstrates a potent synergy with voreloxin in leukemia cell lines and that this effect is associated with the inhibition of proliferation, cell cycle arrest and induction of apoptosis.

We have found that both drugs, either alone or in combination, can inhibit cell growth and the level of this inhibition was dose dependent. The combinations of VX-11e and voreloxin were synergistic over a wide range of concentrations in MOLM-14, REH and MOLT-4 cell lines. In K562 cells, three combinations were found to be additive, one antagonistic and only one synergistic. We have demonstrated that the most synergistic effect of VX-11e and voreloxin combinations occurred in large cell fraction (Fa > 0.7). It has been postulated, that for better therapeutic effect a threshold at Fa = 0.8 (80% of cells affected) should be commonly used when investigating anticancer drugs [23]. In all cell lines tested, ERK has been found to be at high basal level, which is in accordance with the previous data showing that ERK may be strongly activated in leukemia cells [11, 24]. We have shown that only VX-11e significantly reduces ERK activation in leukemia cells and this effect can be still observed after combined treatment with VX-11e and voreloxin.

To further investigate the potential mechanisms of the combined treatment, cell cycle distribution was evaluated. The treatment with VX-11e and voreloxin promoted G0/G1 cell cycle arrest that was associated with the increased expression of cell cycle inhibitor, p21. These results corresponded with the effect of the drugs on the induction of apoptosis. It has been reported that p21 protein is an important regulator of G1 to S phase progression of the cell cycle and its expression is usually induced by p53 protein [25]. Moreover, p21 is considered to play a role in the process of apoptosis [26–28]. On the one hand, a large number of studies have shown that p21 exerts an anti-apoptotic effect in leukemia cells [29–32]. On the other hand, there are evidences showing that some drugs can promote apoptosis through induction of p21 in cancer cells. It was reported that treatment of MCF-7 and MDA-MB-23 breast cancer cell lines with taxol induced an accumulation of cells in G2/M and sub-G1 phase by induction of p21 [33]. Another study demonstrated that ectopic expression of p21 in MCF-7 and T47D cells decreased cell division and promoted apoptosis [34]. Interestingly, in our study, the combination of VX-11e and voreloxin showed increased percentage of apoptotic K562 cells without affecting the G0/G1 arrest and p21 expression. On the basis of the above results, we postulate that this effect could be at least partially related to the absence of p53 protein in K562 cells due to the null mutation [35, 36], whereas MOLM-14 cells have functional p53 [37]. Thus, cells with p53 defect fail to induce p21 expression and activate apoptosis. On the contrary, our data indicate that the increased expression of p21 did not seem to be correlated with p53 status, since p53-mutated REH and MOLT-4 cells overexpressed p21 protein after VX-11e and voreloxin combined treatment [38, 39]. Thus, it is possible that other mechanisms may contribute to the synergistic pro-apoptotic effect of VX-11e and voreloxin in K562 cells and these surprising findings require further investigation.

Survivin, a member of the anti-apoptotic signaling protein family, plays an important role in cell proliferation and survival [40]. Elevated level of survivin has been found in hematological malignancies, including leukemias [41]. Moreover, it was recently reported that in leukemia cells survivin expression was regulated through MEK/ERK-dependent mechanisms [42]. We have demonstrated in the present study that survivin levels are significantly decreased in cells after combinatorial treatment with VX-11e and voreloxin. As shown in other studies on treatment of AML cell lines with inhibitors of MEK/ERK pathway combined with other anticancer drugs, the synergistic pro-apoptotic effect of drugs was accompanied by decreased expression of survivin [43]. Furthermore, previous studies have demonstrated that transcription factors, such as NF-κB, are important for increased survivin transcription activity [44]. It has been shown that activation of the NF-κB signaling pathway contributes to tumor progression by blocking apoptosis via upregulation of survivin [45, 46]. The NF-κB p50 subunit is an active molecule of p105 precursor protein [47]. In the present study, we have observed reduced levels of both p50 and p105 subunits of NF-κB protein in cells treated with both drugs. In addition, we assessed the nuclear staining of NF-κB p105/p50 in leukemia cell lines because the translocation of p50 subunit into the nucleus is one of important steps for transcriptional activation of NF-κB [48]. We found that nuclear staining of NF-κB p105/p50 was reduced after combined VX-11e and voreloxin treatment in all cell lines except K562 cells. These results might suggest that NF-κB pathway is not involved in the anti-proliferative and pro-apoptotic effects in K562 cells. In our study, the combination of VX-11e and voreloxin was synergistic in K562 cells only at concentrations equal to the IC50 values obtained for each drug. The different effectiveness of drugs may reflect the variability of cell lines, as leukemia cells display diverse phenotypes and chromosomal abnormalities. It has been reported that K562 cells carry the Bcr-Abl fusion gene which promotes cell growth, inhibits apoptosis and is responsible for the resistance to different anticancer drugs [49, 50]. Interestingly, NF-κB activation is also dependent on downstream targets of Bcr-Abl including MEK kinase-1 (MEKK1). It has been found that Bcr-Abl enhances MEKK1 expression and kinase activity and strongly induces NF-κB signaling. [51]. Bcr-Abl has been shown to induce activation of NF-κB in LAMA84 cells, a human CML cell line [52]. Furthermore, it has been found, that BCR-ABL fusion protein promotes survivin expression in hematopoietic cells [53].

In conclusion, our study reveals that VX-11e shows synergy with voreloxin in the inhibition of leukemia cell proliferation. The combination of both drugs affects cell cycle and induce apoptosis by enhancing p21 protein expression and decreasing survivin and NF-κB protein levels in leukemia cell lines, with the exception of K562 cells. Although the synergistic effect of VX-11e and voreloxin could depend on the cell type and concentration of drugs, the combination of ERK inhibitors with other anticancer agents seems to be a promising therapeutic strategy for the treatment of leukemia.

## References

[1] Lefloch R, Pouysségur J, Lenormand P (2009). Total ERK1/2 activity regulates cell proliferation. Cell Cycle.

[2] Johannessen M, Delghandi MP, Moens U (2004). What turns CREB on?. Cell Signal.

[3] Chung E, Kondo M (2011). Role of Ras/Raf/MEK/ERK signaling in physiological hematopoiesis and leukemia development. Immunol Res.

[4] Enzler T, Sano Y, Choo MK, Cottam HB, Karin M, Tsao H, Park JM (2011). Cell-selective inhibition of NF-κB signaling improves therapeutic index in a melanoma chemotherapy model. Cancer Discov.

[5] Huang X, Schwind S, Santhanam R, Eisfeld AK, Chiang CL, Lankenau M, Yu B, Hoellerbauer P, Jin Y, Tarighat SS, Khalife J, Walker A, Perrotti D, Bloomfield CD, Wang H, Lee RJ, Lee LJ, Marcucci G (2016). Targeting the RAS/MAPK pathway with miR-181a in acute myeloid leukemia. Oncotarget.

[6] Tyner JW, Erickson H, Deininger MW, Willis SG, Eide CA, Levine RL, Heinrich MC, Gattermann N, Gilliland DG, Druker BJ, Loriaux MM (2009). High-throughput sequencing screen reveals novel, transforming RAS mutations in myeloid leukemia patients. Blood.

[7] Blalock WL, Moye PW, Chang F, Pearce M, Steelman LS, McMahon M, McCubrey JA (2000). Combined effects of aberrant MEK1 activity and BCL2 overexpression on relieving the cytokine dependency of human and murine hematopoietic cells. Leukemia.

[8] Milella M, Kornblau SM, Estrov Z, Carter BZ, Lapillonne H, Harris D, Konopleva M, Zhao S, Estey E, Andreeff M (2001). Therapeutic targeting of the MEK/MAPK signal transduction module in acute myeloid leukemia. J Clin Investig.

[9] Nishioka C, Ikezoe T, Yang J, Yokoyama A (2010). Inhibition of MEK/ERK signaling induces apoptosis of acute myelogenous leukemia cells via inhibition of eukaryotic initiation factor 4E-binding protein 1 and down-regulation of Mcl-1. Apoptosis.

[10] Jain N, Curran E, Iyengar NM, Diaz-Flores E, Kunnavakkam R, Popplewell L, Kirschbaum MH, Karrison T, Erba HP, Green M, Poire X, Koval G, Shannon K, Reddy PL, Joseph L, Atallah EL, Dy P, Thomas SP, Smith SE, Doyle LA, Stadler WM, Larson RA, Stock W, Odenike O (2014). Phase II study of the oral MEK inhibitor selumetinib in advanced acute myelogenous leukemia: a University of Chicago phase II consortium trial. Clin Cancer Res.

[11] Jasek-Gajda E, Gajda M, Jasińska M, Litwin JA, Lis GJ (2018). TAK-733, a selective MEK Inhibitor, enhances voreloxin-induced apoptosis in myeloid leukemia cells. Anticancer Res.

[12] Yap JL, Worlikar S, MacKerell AD, Shapiro P, Fletcher S (2011). Small-molecule inhibitors of the ERK signaling pathway: towards novel anticancer therapeutics. ChemMedChem.

[13] Aronov AM, Tang Q, Martinez-Botella G, Bemis GW, Cao J, Chen G, Ewing NP, Ford PJ, Germann UA, Green J, Hale MR, Jacobs M, Janetka JW, Maltais F, Markland W, Namchuk MN, Nanthakumar S, Poondru S, Straub J, ter Haar E, Xie X (2009). Structure-guided design of potent and selective pyrimidylpyrrole inhibitors of extracellular signal-regulated kinase (ERK) using conformational control. J Med Chem.

[14] Shin M, Franks CE, Hsu KL (2018). Isoform-selective activity-based profiling of ERK signaling. Chem Sci.

[15] Krepler C, Xiao M, Sproesser K, Brafford PA, Shannan B, Beqiri M, Liu Q, Xu W, Garman B, Nathanson KL, Xu X, Karakousis GC, Mills GB, Lu Y, Ahmed TA, Poulikakos PI, Caponigro G, Boehm M, Peters M, Schuchter LM, Weeraratna AT, Herlyn M (2016). Personalized preclinical trials in BRAF inhibitor-resistant patient-derived xenograft models identify second-line combination therapies. Clin Cancer Res.

[16] He L, Tang J, Andersson EI, Timonen S, Koschmieder S, Wennerberg K, Mustjoki S, Aittokallio T (2018). Patient-customized drug combination prediction and testing for T-cell prolymphocytic leukemia patients. Cancer Res.

[17] Hoch U, Lynch J, Sato Y, Kashimoto S, Kajikawa F, Furutani Y, Silverman JA (2009). Voreloxin, formerly SNS-595, has potent activity against a broad panel of cancer cell lines and in vivo tumor models. Cancer Chemother Pharmacol.

[18] Walsby EJ, Coles SJ, Knapper S, Burnett AK (2011). The topoisomerase II inhibitor voreloxin causes cell cycle arrest and apoptosis in myeloid leukemia cells and acts in synergy with cytarabine. Haematologica.

[19] McGowan JV, Chung R, Maulik A, Piotrowska I, Walker JM, Yellon DM (2017). Anthracycline chemotherapy and cardiotoxicity. Cardiovasc Drugs Ther.

[20] Fathi AT, Karp JE (2009). New agents in acute myeloid leukemia: beyond cytarabine and anthracyclines. Curr Oncol Rep.

[21] Sayar H, Bashardoust P (2017). Therapies for acute myeloid leukemia: vosaroxin. Onco Targets Ther.

[22] Chou TC, Talalay P (1984). Quantitative analysis of dose-effect relationships: the combined effects of multiple drugs or enzyme inhibitors. Adv Enzyme Regul.

[23] Chou TC (2010). Drug combination studies and their synergy quantification using the Chou-Talalay method. Cancer Res.

[24] Lunghi P, Tabilio A, Dall’Aglio PP, Ridolo E, Carlo-Stella C, Pelicci PG, Bonati A (2003). Downmodulation of ERK activity inhibits the proliferation and induces the apoptosis of primary acute myelogenous leukemia blasts. Leukemia.

[25] Sherr CJ, Roberts JM (1999). CDK inhibitors: positive and negative regulators of G1-phase progression. Genes Dev.

[26] Chen A, Huang X, Xue Z, Cao D, Huang K, Chen J, Pan Y, Gao Y (2015). The role of p21 in apoptosis, proliferation, cell cycle arrest, and antioxidant activity in UVB-irradiated human HaCaT keratinocytes. Med Sci Monit Basic Res.

[27] Davies C, Hogarth LA, Mackenzie KL, Hall AG, Lock RB (2015). p21(WAF1) modulates drug-induced apoptosis and cell cycle arrest in B-cell precursor acute lymphoblastic leukemia. Cell Cycle.

[28] Baldi A, Piccolo MT, Boccellino MR, Donizetti A, Cardillo I, La Porta R, Quagliuolo L, Spugnini EP, Cordero F, Citro G, Menegozzo M, Calogero RA, Crispi S (2011). Apoptosis induced by piroxicam plus cisplatin combined treatment is triggered by p21 in mesothelioma. PLoS ONE.

[29] Zhang Y, Fujita N, Tsuruo T (1999). Caspase-mediated cleavage of p21Waf1/Cip1 converts cancer cells from growth arrest to undergoing apoptosis. Oncogene.

[30] Chang BD, Watanabe K, Broude EV, Fang J, Poole JC, Kalinichenko TV, Roninson IB (2000). Effects of p21Waf1/Cip1/Sdi1 on cellular gene expression: implications for carcinogenesis, senescence, and age-related diseases. Proc Natl Acad Sci USA.

[31] Wu X, Yang N, Zhou WH, Xu J, Chen JJ, Zheng FM, Long ZJ, Yue CF, Ai KX, Liu LL, Wan XY, Liu Q (2014). Up-regulation of P21 inhibits TRAIL-mediated extrinsic apoptosis, contributing resistance to SAHA in acute myeloid leukemia cells. Cell Physiol Biochem.

[32] Javelaud D, Besancon F (2002). Inactivation of p21WAF1 sensitizes cells to apoptosis via an increase of both p14ARF and p53 levels and an alteration of the Bax/Bcl-2 ratio. J Biol Chem.

[33] Choi YH, Yoo YH (2012). Taxol-induced growth arrest and apoptosis is associated with the upregulation of the Cdk inhibitor, p21WAF1/CIP1, in human breast cancer cells. Oncol Rep.

[34] Sheikh MS, Rochefort H, Garcia M (1995). Overexpression of p21WAF1/CIP1 induces growth arrest, giant cell formation and apoptosis in human breast carcinoma cell lines. Oncogene.

[35] Li Y, Zhao K, Yao C, Kahwash S, Tang Y, Zhang G, Patterson K, Wang QE, Zhao W (2016). Givinostat, a type II histone deacetylase inhibitor, induces potent caspase-dependent apoptosis in human lymphoblastic leukemia. Genes Cancer.

[36] Mobaraki RN, Karimi M, Alikarami F, Farhadi E, Amini A, Bashash D, Paridar M, Kokhaei P, Rezvani MR, Kazemi A, Safa M (2018). RITA induces apoptosis in p53-null K562 leukemia cells by inhibiting STAT5, Akt, and NF-κB signaling pathways. Anticancer Drugs.

[37] Weisberg E, Halilovic E, Cooke VG, Nonami A, Ren T, Sanda T, Simkin I, Yuan J, Antonakos B, Barys L, Ito M, Stone R, Galinsky I, Cowens K, Nelson E, Sattler M, Jeay S, Wuerthner JU, McDonough SM, Wiesmann M, Griffin JD (2015). Inhibition of wild-type p53-expressing AML by the novel small molecule HDM2 inhibitor CGM097. Mol Cancer Ther.

[38] Trino S, Iacobucci I, Erriquez D, Laurenzana I, De Luca L, Ferrari A, Ghelli Luserna Di Rorà A, Papayannidis C, Derenzini E, Simonetti G, Lonetti A, Venturi C, Cattina F, Ottaviani E, Abbenante MC, Russo D, Perini G, Musto P, Martinelli G (2016). Targeting the p53 MDM2 interaction by the small-molecule MDM2 antagonist Nutlin-3a: a new challenged target therapy in adult Philadelphia positive acute lymphoblastic leukemia patients. Oncotarget.

[39] Bhatia U, Danishefsky K, Traganos F, Darzynkiewicz Z (1995). Induction of apoptosis and cell cycle-specific change in expression of p53 in normal lymphocytes and MOLT-4 leukemic cells by nitrogen mustard. Clin Cancer Res.

[40] Li D, Hu C, Li H (2018). Survivin as a novel target protein for reducing the proliferation of cancer cells. Biomed Rep.

[41] Huang J, Lyu H, Wang J, Liu B (2015). Influence of survivin-targeted therapy on chemosensitivity in the treatment of acute myeloid leukemia. Cancer Lett.

[42] Carter BZ, Milella M, Altieri DC, Andreeff M (2001). Cytokine-regulated expression of survivin in myeloid leukemia. Blood.

[43] Zhang W, Ruvolo VR, Gao C, Zhou L, Bornmann W, Tsao T, Schober WD, Smith P, Guichard S, Konopleva M, Andreeff M (2014). Evaluation of apoptosis induction by concomitant inhibition of MEK, mTOR, and Bcl-2 in human acute myelogenous leukemia cells. Mol Cancer Ther.

[44] Kawakami H, Tomita M, Matsuda T, Ohta T, Tanaka Y, Fujii M, Hatano M, Tokuhisa T, Mori N (2005). Transcriptional activation of survivin through the NF-kappaB pathway by human T-cell leukemia virus type I tax. Int J Cancer.

[45] Takizawa BT, Uchio EM, Cohen JJ, Wheeler MA, Weiss RM (2007). Downregulation of survivin is associated with reductions in TNF receptors’ mRNA and protein and alterations in nuclear factor kappa B signaling in urothelial cancer cells. Cancer Investig.

[46] Schreck R, Albermann K, Baeuerle PA (1992). Nuclear factor kappa B: an oxidative stress-responsive transcription factor of eukaryotic cells (a review). Free Radic Res Commun.

[47] Liou HC, Nolan GP, Ghosh S, Fujita T, Baltimore D (1992). The NF-kappa B p50 precursor, p105, contains an internal I kappa B-like inhibitor that preferentially inhibits p50. EMBO J.

[48] Gao K, Dai DL, Martinka M, Li G (2006). Prognostic significance of nuclear factor-kappaB p105/p50 in human melanoma and its role in cell migration. Cancer Res.

[49] Lozzio CB, Lozzio BB (1975). Human chronic myelogenous leukemia cell-line with positive Philadelphia chromosome. Blood.

[50] O’Hare T, Eide CA, Deininger MW (2007). Bcr-Abl kinase domain mutations, drug resistance, and the road to a cure for chronic myeloid leukemia. Blood.

[51] Nawata R, Yujiri T, Nakamura Y, Ariyoshi K, Takahashi T, Sato Y, Oka Y, Tanizawa Y (2003). MEK kinase 1 mediates the antiapoptotic effect of the Bcr-Abl oncogene through NF-kappaB activation. Oncogene.

[52] Carrà G, Torti D, Crivellaro S, Panuzzo C, Taulli R, Cilloni D, Guerrasio A, Saglio G, Morotti A (2016). The BCR-ABL/NF-κB signal transduction network: a long lasting relationship in Philadelphia positive Leukemias. Oncotarget.

[53] Fang ZH, Dong CL, Chen Z, Zhou B, Liu N, Lan HF, Liang L, Liao WB, Zhang L, Han ZC (2009). Transcriptional regulation of survivin by c-Myc in BCR/ABL-transformed cells: implications in anti-leukaemic strategy. J Cell Mol Med.

